# Hepatic Macrophage Abundance and Phenotype in Aging and Liver Iron Accumulation

**DOI:** 10.3390/ijms23126502

**Published:** 2022-06-10

**Authors:** Steven A. Bloomer

**Affiliations:** Division of Science and Engineering, Penn State Abington, 1600 Woodland Rd, Abington, PA 19001, USA; sab320@psu.edu

**Keywords:** aging, deferoxamine, hemosiderosis, macrophage polarization

## Abstract

Liver macrophages serve important roles in iron homeostasis through phagocytosis of effete erythrocytes and the export of iron into the circulation. Conversely, intracellular iron can alter macrophage phenotype. Aging increases hepatic macrophage number and nonparenchymal iron, yet it is unknown whether age-related iron accumulation alters macrophage number or phenotype. To evaluate macrophages in a physiological model of iron loading that mimicked biological aging, young (6 mo) Fischer 344 rats were given one injection of iron dextran (15 mg/kg), and macrophage number and phenotype were evaluated via immunohistochemistry. A separate group of old (24 mo) rats was treated with 200 mg/kg deferoxamine every 12 h for 4 days. Iron administration to young rats resulted in iron concentrations that matched the values and pattern of tissue iron deposition observed in aged animals; however, iron did not alter macrophage number or phenotype. Aging resulted in significantly greater numbers of M1 (CD68^+^) and M2 (CD163^+^) macrophages in the liver, but neither macrophage number nor phenotype were affected by deferoxamine. Double-staining experiments demonstrated that both M1 (iNOS^+^) and M2 (CD163^+^) macrophages contained hemosiderin, suggesting that macrophages of both phenotypes stored iron. These results also suggest that age-related conditions other than iron excess are responsible for the accumulation of hepatic macrophages with aging.

## 1. Introduction

In addition to their well-known immunological functions, hepatic macrophages play important roles in overall homeostasis. They are crucial for whole-body iron turnover due to their role in the phagocytosis of senescent red blood cells and the export of the liberated iron back into the circulation. Hepatic macrophages express transferrin receptor-1 (TFR1), which imports transferrin-bound iron from the circulation via receptor-mediated endocytosis [1]. Additionally, divalent metal transporter-1 (DMT1) mediates uptake of non-transferrin-bound iron [1]. The imported iron is then brought into the acidic lysosomal compartment, after which it can be delivered to organelles such as the mitochondria, where it is incorporated into iron–sulfur centers in the electron transport chain or heme-containing enzymes. Iron not immediately utilized for these purposes can be stored in the iron storage protein, ferritin. In cases of more severe iron overload, ferritin stores can be overwhelmed, which results in the formation of the long-term iron storage compartment, hemosiderin [2]. Under conditions of physiological iron demand (i.e., erythropoiesis), iron can be mobilized from both storage compartments and exits the cell via the iron exporter, ferroportin, which is highly expressed on hepatic macrophages [3].

Generally, macrophages exist on a spectrum between two phenotypes: the pro-inflammatory M1 phenotype and the anti-inflammatory/matrix remodeling M2 phenotype. The shifting of macrophage phenotype between these two polarizations depends on signals from the extracellular milieu. Interestingly, recent studies have identified differences in iron handling and iron regulatory protein expression between these phenotypes. For example, macrophages polarized to the M1 phenotype exhibited a greater abundance of the iron storage protein, ferritin, than M2 macrophages, likely reflecting their role in inflammation-induced iron sequestration [4,5]. Conversely, M2 macrophage polarization in mouse bone marrow-derived macrophages and human monocytes resulted in greater expression of the iron exporter, ferroportin, and greater iron release than M1 macrophages [4,5].

Polarization affects cellular iron handling and vice versa–both iron chelation and iron addition modulate the M1 phenotype. An early study in this field demonstrated that in vivo chelation of iron decreased NFκB activity in isolated rat hepatic macrophages. In that study, the authors also showed that short-term iron chelation of primary hepatic macrophages suppressed LPS-induced expression of TNFα and IL-6 [6]. The authors confirmed the role of iron in inflammatory activation by demonstrating that giving iron back after chelation restored NFκB activity in isolated macrophages [6]. The inhibition of NFκB by the antioxidant N-acetylcysteine (NAC) suggested that iron-induced NFκB activation occurred through stimulating a pro-oxidative environment [6]. More recently, Pereira and associates demonstrated in human monocyte-derived macrophages that the iron chelator, deferoxamine, prevented LPS-induced TNFα induction [7]. Other studies have treated isolated macrophages with various forms of iron. Handa and associates demonstrated that ferric ammonium citrate (FAC; 4 h treatment with 250 μM) stimulated the expression of iNOS, IL-6, and TNFα in bone marrow-derived macrophages [8]. Similarly, Zhou et al. observed an increase in iNOS and TNFα after treatment of RAW264.7 mouse macrophages with ferric citrate or ferrous sulfate (2500 μg/mL for 2 h), with no change in M2 markers [9].

On the contrary, studies have demonstrated that treatment of macrophages with iron induces the opposite, M2 polarization. When treated with ferrous sulfate (100 µM for 7 days), macrophages derived from the human monocytic leukemia cell line (THP-1) demonstrated attenuated expression of the M1 marker, iNOS, along with greater expression of the M2 markers, CD163 and CD206. The authors of that study repeated the latter result in bone-marrow-derived macrophages from mice [10]. Similarly, Gan et al. observed that FAC (25 µg/mL for 24 h) lowered the expression of iNOS, IL-6, and TNFα in M1-polarized mouse RAW264.7 macrophages [11]. Further, they demonstrated that iron treatment lowered the activity of STAT1, an important transcription factor in pro-inflammatory cytokine production, which suggests a likely mechanism for the attenuation of the M1 response in their model [11]. Thus, it appears that short-term incubation with high concentrations of iron results in M1 polarization, while longer incubations with lower concentrations of iron result in M2 polarization. These lower concentrations of iron better simulate the concentrations that macrophages would experience in vivo [12,13]. These differing outcomes raise the need to further evaluate the role of iron in macrophage polarization, particularly in an in vivo model.

Given the results in cell culture experiments, it was of interest to develop a physiologically relevant model of iron loading and then evaluate macrophage phenotype. For this purpose, young (6 month-old) Fischer 344 rats were treated with iron dextran, a modality that, in relatively low doses, causes iron deposition primarily in hepatic macrophages. This iron deposition mimics what has been observed in aged rats [14], and aging is associated with an increase in liver macrophage number [15,16,17,18]. However, it is unknown whether the excess iron serves as the proliferative stimulus [19], leading to a greater number of macrophages. Therefore, the goals of this study were two-fold: (1) to determine whether iron loading shifts macrophage phenotype in vivo and (2) to determine whether greater macrophage iron (similar to that observed in old rats) results in a greater number of macrophages. It was hypothesized that iron treatment would shift the macrophage phenotype to the M2 polarization and result in a greater number of macrophages in the livers of young animals.

## 2. Results

### 2.1. Liver Iron Deposition

**Figure 1:**
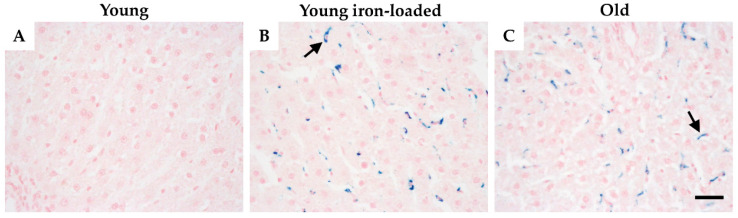
Treatment with iron-dextran increases hepatic iron deposition in young rats. Liver sections were stained with the Perls’ Prussian Blue method for ferric iron, and images from the 40× objective are shown. Punctate blue spots indicate nonparenchymal iron accumulation, which was uniformly absent from young control animals (**A**) but evident in the iron-treated young animals (**B**) and in the old animals (**C**). Note the similarity of the pattern of distribution of iron between young, iron-treated, and old animals. Images are representative of 6 animals per group. Arrows indicate hemosiderin deposits in each group. Scale bar is 50 μm.

Treatment of young animals with iron-dextran (15 mg/kg) increased hepatic iron concentration 2-fold (to values observed in old animals; Table 1) and resulted in an identical pattern of nonparenchymal iron deposition as observed in old rats (Figure 1). The positive Perls’ reaction in nonparenchymal cells indicates that iron accumulated as hemosiderin in macrophages. Hemosiderin typically accumulates when cellular ferritin stores have become overwhelmed [2,20]; therefore, the iron loading protocol resulted in hemosiderosis, which exactly mimicked the iron deposition observed in old animals. Treatment of aged animals with deferoxamine (DFO) lowered hepatic iron but did not eliminate nonparenchymal iron deposition or affect macrophage number or polarization (Figure S1).

### 2.2. Assessment of Iron Loading and Aging on M1 Hepatic Macrophages

**Figure 2:**
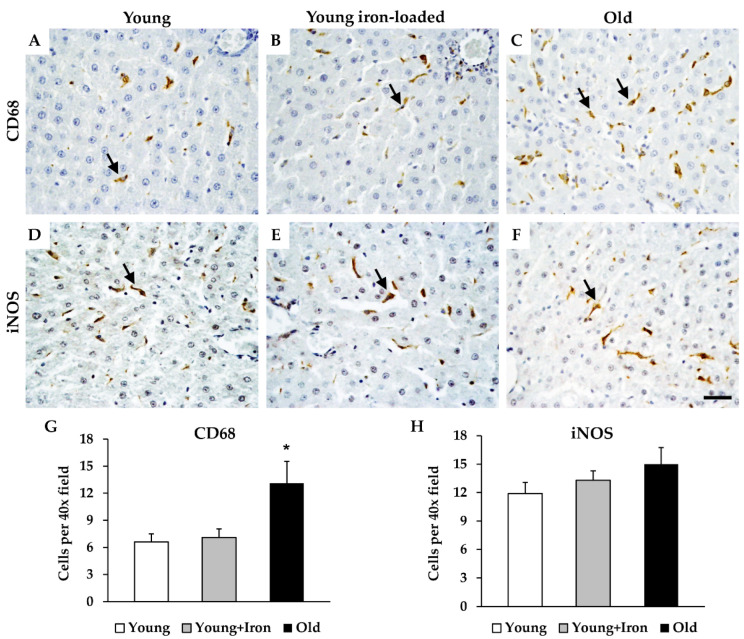
Immunohistochemistry for putative M1 macrophage markers in the liver with iron loading and aging. Liver sections were stained for either CD68 (**A**–**C**) or iNOS (**D**–**F**) in young control (**A**,**D**), young, iron-loaded (**B**,**E**), and old animals (**C**,**F**). Quantification of CD68^+^ (**G**) and iNOS^+^ (**H**) cells was expressed as counts per 40× micrographic field. Arrows indicate cells positive for each marker. Data are mean (+SEM) cell counts per field; 8 fields were counted per animal (*n* = 6 per group). * Significant difference between aged animals and both young groups. Scale bar is 50 μm.

Treatment of young animals with iron dextran did not alter either the number of CD68^+^ or iNOS^+^ cells (Figure 2). However, aged animals demonstrated a significantly greater number of CD68^+^ macrophages (*p* < 0.017), and a trend for an increase in iNOS^+^ macrophages (*p* = 0.09; Figure 2).

### 2.3. Assessment of Iron Loading and Aging on M2 Hepatic Macrophages

**Figure 3:**
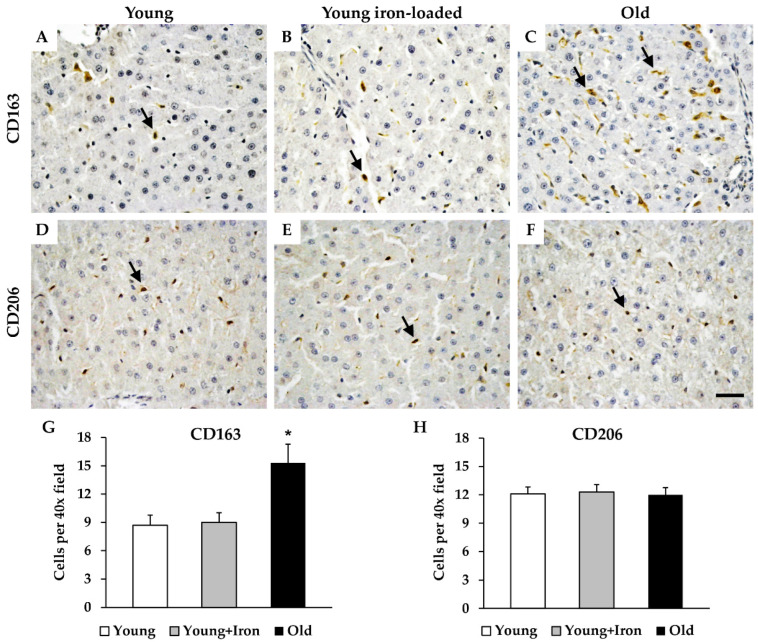
Evaluation of putative M2 macrophage markers in the liver with iron loading and aging. Liver sections were stained for either CD163 (**A**–**C**), or CD206 (**D**–**F**) in young control (**A**,**D**), young, iron-loaded (**B**,**E**), and old animals (**C**,**F**). Quantification of CD163^+^ (**G**) and CD206^+^ (**H**) cells was expressed as counts per 40× micrographic field. Arrows indicate cells positive for each marker. Data are mean (+SEM) cell counts per field; 8 fields were counted per animal (*n* = 6 per group). * Significant difference between aged animals and both young groups. Scale bar is 50 μm.

Similar to the M1 markers, iron loading did not alter the number of CD163^+^ or CD206^+^ cells in young animals. One subset of M2 macrophages, CD163^+^ cells, was significantly elevated in aged animals compared to young animals; however, the number of CD206^+^ cells was similar between young and old animals (Figure 3).

### 2.4. Macrophage Number and Polarization in Young Animals Treated with Iron Dextran

**Figure 4:**
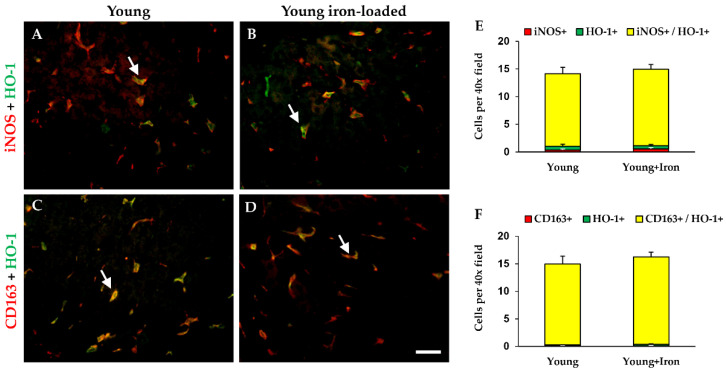
Assessment of macrophage polarization with iron loading in young rats (6 mo). Liver sections were double-stained for iNOS and HO-1 (M1 polarization; **A**,**B**) and CD163 and HO-1 (M2 polarization; **C**,**D**) in young control (**A**,**C**) and young, iron-loaded animals (**B**,**D**). Quantitation of cells positive for each marker alone and double-positive for both markers were performed. Quantitation of M1 macrophages is shown in panel (**E**) and M2 macrophages in panel (**F**). Data are mean (+SEM) cell counts per field; 5 separate fields were counted per animal (*n* = 6 per group). Scale bar is 50 μm.

Double-labeling experiments did not demonstrate a shift in polarization with iron treatment (Figure 4); similar numbers of M1 (iNOS^+^/HO-1^+^) and M2 (CD163^+^/HO-1^+^) macrophages were observed between young and young iron-treated animals. As demonstrated previously [15], the majority of macrophages in each group were positive for both the polarization marker (iNOS or CD163) and HO-1 (Figure 4). The percentage of iNOS^+^/HO-1^+^ cells in young and young iron-administered rats was 92.3% and 92.9%, respectively (not significant). In young and young, iron-treated animals, the percentages of CD163^+^/HO-1^+^ cells were 98.5% and 97%, respectively (not significant). It should be noted that this same labeling technique successfully detected an alteration in polarization in response to gadolinium chloride in aged rats in a previous investigation [15].

### 2.5. Iron Accumulation in M1 and M2 Macrophages

**Figure 5:**
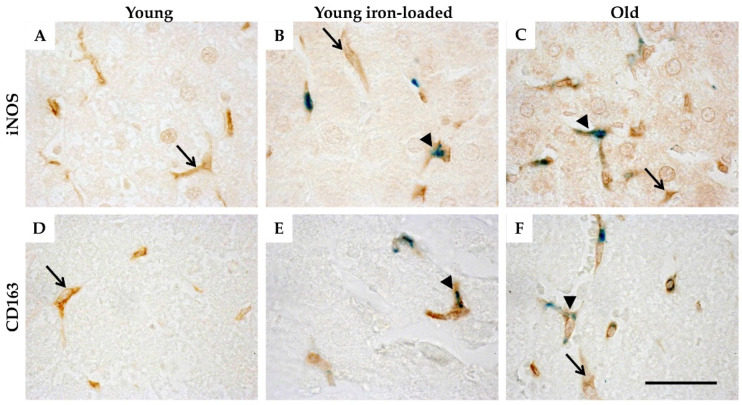
M1 and M2 macrophages exhibit iron deposition. Samples were stained for either the M1 marker, iNOS (**A**–**C**), or the M2 marker, CD163 (**D**–**F**), and afterward for ferric iron (Perls’ staining) in young control (**A**,**D**), young, iron-loaded (**B**,**E**), and old animals (**C**,**F**). Double-positive cells (arrowheads) appear brown with green inclusions. Some macrophages in iron-treated young animals and old animals do not stain positive for iron (arrows). Images are representative of 6 animals per group. Scale bar is 50 μm.

Finally, both M1 and M2 macrophages exhibited iron accumulation in young rats treated with iron and in aged animals, although some macrophages lacked iron deposits (Figure 5). Quantitation of the relative numbers of iron-positive M1 and M2 cells was not performed because the highly acidic conditions of the Perls’ reaction interferes with DAB staining [21]; this was also observed in the present investigation.

## 3. Discussion

This investigation provides novel insights into the biology of macrophages. Specifically, short-term iron loading in young rats that exactly mimicked iron concentrations and the pattern of deposition in old rats did not alter macrophage number or phenotype. This suggests that the excess iron deposition alone in aged rats might not be responsible for the greater number of macrophages observed in this age group. However, given that aging results in macrophage iron accrual, it cannot be ruled out that long-term changes in iron metabolism affect macrophages. Indeed, a previous study found greater labile iron (“free” or “reactive” iron) in the aged rat liver [22]. Since that study analyzed whole-tissue homogenates, its results reflect labile iron primarily in hepatocytes, which comprise the majority of liver cells [23]. A more recent study demonstrated an age-related increase in labile iron in human monocytes, which can differentiate into tissue macrophages [24]. Taken together, these results suggest that the labile iron pool could also be elevated in aged hepatic macrophages. Iron can mediate a pro-oxidative environment leading to the stimulation of pro-inflammatory cytokines such as IL-6 and TNFα, which enhance macrophage proliferation [6,25]. Therefore, changes in macrophage number might require longer-term changes in iron than were investigated here. The combination of excess iron and the aging tissue milieu could predispose to changes in macrophage function [14]. Future studies should test whether longer-term treatment with a more cell-permeable iron chelator such as deferiprone would effectively decrease iron in this compartment and alter macrophage biology. Overall, what leads to the accumulation of macrophages in the aged liver remains an open question, but it could be due to chronic changes in the redox or inflammatory environment that accompanies aging (reviewed in [14]).

With respect to macrophage markers, the results of this investigation are consistent with previous observations showing an increase in M1 and M2 macrophage markers with aging [15]. However, it should be noted that while some investigators have used CD68 as an M1 marker [8,26], others consider it a more general marker of tissue macrophages [27,28,29]. Interestingly, CD206^+^ cells did not change with aging. This observation can be explained by the fact that both macrophages and endothelial cells express CD206 in the liver [30]. With aging, there is an increase in M2 macrophages (Figure 3 and [15]) but a decrease in endothelial cell-specific gene expression (eNOS; [17]) which could explain the similar numbers of CD206^+^ cells between age groups.

While many investigations on macrophage polarization have focused on isolated macrophages, fewer have focused on macrophage function in vivo. Recently, Agoro and associates evaluated macrophage polarization markers in the livers of C57BL/6 mice after a single intraperitoneal injection of 2000 mg/kg iron dextran, which resulted in a greater than 120-fold increase in hepatic iron concentrations. In liver homogenates, they observed a 40-fold increase in the M2 marker, Ym1, but no change in the M2 marker, arginase-1, at 48 h after iron injection. Also, they observed an approximately 6-fold increase in the M1 marker, iNOS. In that study, peritoneal macrophages isolated from the iron-treated mice also exhibited significant increases in Ym1 and iNOS [31]. Their results demonstrate that very high doses of iron are required to increase both M1 and M2 markers in the liver. However, since macrophages were not counted, it is unknown whether their results represented a change in marker expression or the number of tissue macrophages. The increases in macrophage markers and iron in their study resemble the aging phenotype, yet the hepatic iron concentrations are far in excess of those observed in old animals (~23,000 vs. ~800 μg/gram dry weight [31,32]).

In a model of chronic, progressive iron overload induced by iron dextran, hepatic iron concentrations were elevated greater than 50-fold, with the excess iron deposited in both hepatocytes and nonparenchymal cells [33]. In the iron-treated group, the authors observed nonparenchymal cells that stained positive for the proliferation marker, proliferating cell nuclear antigen (PCNA). While these cells were rare, they did not observe nonparenchymal PCNA staining in control animals [33]. The 50-fold increase in iron was also associated with a 3-fold increase in whole-liver TNFα expression, which can stimulate macrophage proliferation [25]. These results suggest that iron overload can stimulate proliferation in nonparenchymal cells (likely macrophages) but that supraphysiological concentrations of iron are needed for this to occur. The current study demonstrates that the presence of excess iron in hepatic macrophages in concentrations more physiologically relevant to the aging phenotype does not cause a change in macrophage number or phenotype. Because a 5-day time point was evaluated in the current investigation, it is possible that early changes in macrophage phenotype were missed; therefore, it will be important to determine whether there are short-term changes in macrophage number or phenotype in this model of iron loading. 

The current investigation demonstrated that both M1 and M2 macrophages stored iron in the form of hemosiderin in both iron-treated young rats and aged rats. This storage of excess iron in CD163^+^ macrophages conflicts somewhat with the iron-export phenotype of M2 macrophages observed in cell culture studies from bone marrow-derived macrophages [4,5]. This may reflect the different extracellular niches [34], with the bone marrow favoring macrophage iron efflux and the liver favoring storage. The liver expresses greater amounts of hepcidin than the bone marrow [35,36]. Since hepcidin blocks iron efflux from macrophages, stronger paracrine suppression of iron export from macrophages likely exists in the liver compared to the bone marrow. Also, cell isolation procedures and subsequent culture affect cellular phenotype and macrophage polarization [37,38] which could explain differences in iron handling between the in vivo work described here and the cell culture experiments.

## 4. Materials and Methods

### 4.1. Animal Protocols

All animal protocols were conducted at the University of Iowa in the laboratory of Dr. Kevin Kregel under IACUC protocol 0606117 and are consistent with the National Research Council’s Guide for the Care and Use of Laboratory Animals. The animal samples utilized in this investigation are the formalin-fixed samples from a previous investigation [32] and are distinct from those utilized in a separate study on aging and hepatic macrophages [15]. Young (6 months) and old (24 months) Fischer 344 rats were obtained from the National Institutes on Aging. Three groups of animals (*n* = 6 per group) were evaluated in this study: young saline-treated; young iron-treated; and old saline-treated. After performing pilot studies using various concentrations of iron dextran injections in young rats (10, 15, 20, and 50 mg/kg), a dose of 15 mg/kg resulted in similar hepatic iron concentrations (HICs) and a similar pattern of tissue distribution as old rats; thus, the young iron-treated group was injected with a single dose of 15 mg/kg iron dextran. Control young animals received a similar dose of dextran in saline. In old rats, preliminary work utilized deferoxamine injections (DFO; 200 mg/kg every 12 h for 4 days) to lower hepatic iron. This treatment caused a subtle but significant decrease in HIC, along with a nearly 6-fold increase in transferrin receptor-1 expression, confirming an appropriate physiological response to the iron chelator [32]. Control animals received isovolumetric doses of 0.9% saline. Animals were euthanized under pentobarbital sodium 5 days after the dose of iron dextran or 2 h after the last dose of DFO.

### 4.2. Nonheme Iron

Hepatic nonheme iron content (HIC) was determined spectrophotometrically by the method of Torrance and Bothwell, as described previously [32]. The HICs in Table 1 have been reported in an earlier investigation [32] and are used with permission.

### 4.3. Histological Protocols

Liver samples were fixed overnight in 10% neutral buffered formalin, dehydrated in a graded series of ethanols, incubated in xylene substitute overnight, and then embedded in paraffin wax. Tissue sections (5 µm) were affixed to Superfrost slides, incubated in xylenes, and then rehydrated with decreasing concentrations of ethanol. To evaluate iron deposition, sections were stained utilizing the Perls’ Prussian Blue protocol. Briefly, rehydrated sections were stained for 20 min in a solution of 10% HCl and 5% potassium ferrocyanide and then rinsed with water. Sections were counterstained with Nuclear Fast Red for 2 min, rinsed with water, dehydrated in increasing concentrations of ethanol, cleared with xylenes, and then cover-slipped with Permount.

### 4.4. Immunohistochemistry

Immunohistochemistry for M1 markers (iNOS and CD68) and M2 markers (CD163 and CD206) was performed utilizing the diaminobenzidine (DAB) colorimetric protocol as described previously [39]. The concentrations of primary antibodies were as follows: iNOS (Abcam #ab15323; 1:10; 5.2 μg/mL); CD68 (BioRad # MCA341R; 1:100; 10 μg/mL); CD163 (Abcam # ab182422, 1:100, 7 μg/mL); and CD206 (Abcam #ab64693; 1:250; 3.6 μg/mL). To distinguish positive staining, negative controls were included on each slide by using nonspecific antisera. With the exception of CD68, these antibodies were used in a previous study [15] and have all been validated by their manufacturers. The pattern of staining of all antigens observed in the present study was consistent with data published in the Human Protein Atlas (www.proteinatlas.org accessed on 14 May 2022; [40]). The biotin-conjugated secondary antibodies were from Vector and used at a dilution of 1:200 (2.5 µg/mL mouse; 7.5 µg/mL rabbit).

Double-immunofluorescence for M1 macrophages (iNOS + HO-1) and M2 macrophages (CD163 + HO-1) was performed as described previously [15]. Unlike F4-80 in the mouse, there is not a well-accepted “pan” macrophage marker for rats. However, two investigations have demonstrated that HO-1 is expressed in the quiescent liver only by macrophages and not hepatocytes, endothelial cells, or stellate cells [41,42]. Thus, nonparenchymal cells positive for both iNOS and HO-1 were considered M1, and cells positive for CD163 and HO-1 were considered M2 macrophages. As demonstrated previously [15] and in this investigation, very few macrophages were positive for only one marker.

To evaluate iron deposition and macrophage polarization simultaneously, separate slides were stained for either iNOS (M1) or CD163 (M2) and then incubated in the Perls’ reagent for 20 min but not counterstained.

### 4.5. Microscopy

Microscopic images of the periportal liver region were taken with a Nikon DXM1200F digital camera using the 40× objective of a Nikon Eclipse E800 microscope. Fluorescent images were generated with the Nikon VFM epifluorescence attachment. ACT-1 (v 2.51) software was utilized to capture images. Nonparenchymal cells positive for each marker were counted in a blind fashion in 8 micrographs per animal. Brightfield images of double-staining for iron deposition and macrophage polarization were taken at the 1000× magnification.

### 4.6. Statistics

The mean number of positive cells per field was compared by ANOVA. When first indicated significant by the ANOVA (a *p*-value of less than 0.05), *t*-tests were performed between groups. A Bonferroni adjustment (0.05/# of groups) was utilized to determine significance, and results were considered significant below an adjusted *p*-value of 0.017.

## 5. Conclusions

Using two immunological markers of the M1 polarization and two markers of the M2 polarization, this study has demonstrated that an iron loading protocol sufficient to cause age-related hemosiderosis in young animals did not modify macrophage number or phenotype. Interestingly, both M1 and M2 macrophages stored hemosiderin; thus, this study provides novel insights into hepatic macrophage function—specifically, their ability to take up and store excess iron without proliferating or shifting their phenotype.

## Figures and Tables

**Table 1:** Hepatic iron concentrations (HIC) in each animal group.

Animal Group	HIC (μg/g)
Young, saline-treated	392.1 ± 14.5 ^a^
Young, iron-treated	811.2 ± 34.2 ^b^
Old, saline-treated	845.4 ± 31.4 ^b^
Old, DFO-treated	696.7 ± 26.6 ^c^

Data are means ± SEM. DFO: Deferoxamine. Groups with different letters are significantly different from each other (*p* < 0.05).

## Data Availability

The data presented in this study are available on request from the corresponding author.

## Supplementary Materials

The following supporting information can be downloaded at: https://www.mdpi.com/article/10.3390/ijms23126502/s1.
