# Supplementary material for: Hepatic Macrophage Abundance and Phenotype in Aging and Liver Iron Accumulation
Source: Int J Mol Sci. 2022 Jun 10;23(12):6502. doi: 10.3390/ijms23126502 (PMC9223835; doi:10.3390/ijms23126502)
Supplement: Supplementary file 1 [file ijms-23-06502-s001.zip › ijms-1755024-supplementary.pdf]

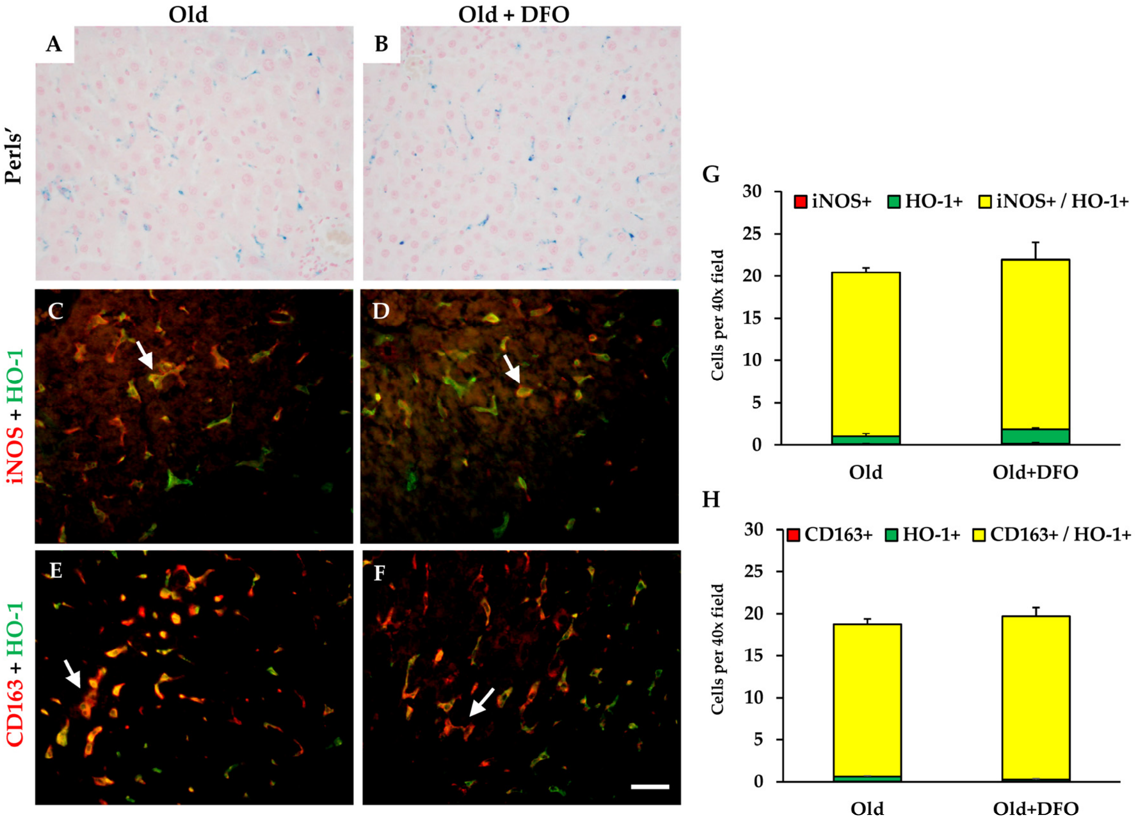

Figure S1. Assessment of macrophage polarization with iron chelation in old (24 mo) rats. Liver sections of old control (A, C, and E) and old, deferoxamine-treated (B,D, and F) rats were stained for iron with Perls' Prussian Blue (A and B). Sections from each group were double-stained for iNOS and HO-1 (M1 polarization; C and D) and CD163 and HO-1 (M2 polarization; E and F). Quantitation of M1 macrophages are shown in panel G and M2 macrophages in panel H. Data are mean (+SEM) cell counts per field; 8 fields were counted per animal (n=6 per group). Scale bar is 50 μm.
